# Regulation of Human Macrophage M1–M2 Polarization Balance by Hypoxia and the Triggering Receptor Expressed on Myeloid Cells-1

**DOI:** 10.3389/fimmu.2017.01097

**Published:** 2017-09-07

**Authors:** Federica Raggi, Simone Pelassa, Daniele Pierobon, Federica Penco, Marco Gattorno, Francesco Novelli, Alessandra Eva, Luigi Varesio, Mirella Giovarelli, Maria Carla Bosco

**Affiliations:** ^1^Laboratory of Molecular Biology, Giannina Gaslini Institute, Genoa, Italy; ^2^Department of Molecular Biotechnology and Health Sciences, University of Turin, Center for Experimental Research and Medical Studies (CERMS), AOU Città della Salute e della Scienza di Torino, Turin, Italy; ^3^Pediatria II, Department of Pediatrics, Giannina Gaslini Institute, University of Genoa, Genoa, Italy

**Keywords:** macrophages, hypoxia, polarization, immunoregulatory receptors, inflammation

## Abstract

Macrophages (Mf) are a heterogeneous population of tissue-resident professional phagocytes and a major component of the leukocyte infiltrate at sites of inflammation, infection, and tumor growth. They can undergo diverse forms of activation in response to environmental factors, polarizing into specialized functional subsets. A common hallmark of the pathologic environment is represented by hypoxia. The impact of hypoxia on human Mf polarization has not been fully established. The objective of this study was to elucidate the effects of a hypoxic environment reflecting that occurring *in vivo* in diseased tissues on the ability of human Mf to polarize into classically activated (proinflammatory M1) and alternatively activated (anti-inflammatory M2) subsets. We present data showing that hypoxia hinders Mf polarization toward the M1 phenotype by decreasing the expression of T cell costimulatory molecules and chemokine homing receptors and the production of proinflammatory, Th1-priming cytokines typical of classical activation, while promoting their acquisition of phenotypic and secretory features of alternative activation. Furthermore, we identify the triggering receptor expressed on myeloid cells (TREM)-1, a member of the Ig-like immunoregulatory receptor family, as a hypoxia-inducible gene in Mf and demonstrate that its engagement by an agonist Ab reverses the M2-polarizing effect of hypoxia imparting a M1-skewed phenotype to Mf. Finally, we provide evidence that Mf infiltrating the inflamed hypoxic joints of children affected by oligoarticular juvenile idiopatic arthritis express high surface levels of TREM-1 associated with predominant M1 polarization and suggest the potential of this molecule in driving M1 proinflammatory reprogramming in the hypoxic synovial environment.

## Introduction

Macrophages (Mf) are a heterogeneous population of tissue-resident professional phagocytes and antigen-presenting cells and represent a major component of the leukocyte infiltrate at sites of inflammation, infection, tissue damage, and tumor growth, originating from the terminal differentiation of circulating monocytes (Mn) (1, 2). They are central effector cells in host defense to pathogens and play key roles in the orchestration of both innate and adaptive immune responses and the regulation of tissue remodeling and repair (3, 4). However, they are also critically involved in the pathogenesis of several chronic inflammatory, autoimmune, and parasitic diseases and in tumor progression (4, 5). Mf are highly versatile cells, able to display distinct phenotypes and functional programs in different tissues (1, 3, 6). Such heterogeneity depends on their ability to undergo different forms of activation in response to distinct signals, including cytokines, damage- and pathogen-associated molecular patterns (DAMPs/PAMPs), and metabolites (6–8). The nature of the activating stimulus and the combination of different stimuli can profoundly impact upon the type of response that occurs, polarizing Mf into specialized functional subsets (3, 6, 9).

Two main subsets of polarized Mf have been defined based on the type of *in vitro* stimulation, surface molecule expression pattern, secretory profile, and functional properties, which mirror the Th1/Th2 dichotomy and represent the extremes of a continuum of activation states: the classically activated (type 1 proinflammatory or M1) and the alternatively activated (type 2 anti-inflammatory or M2) Mf (1, 8–14). M1-polarized Mf originate in response to stimulation with microbial factors, like LPS, and Th1 proinflammatory cytokines, such as IFNγ, TNFα, and IL-1β, or a combination of the two (10, 15), whereas the M2 subset comprises various forms of non-classically activated Mf originating from exposure to different stimuli, such as the Th2 cytokines, IL-4, or IL-13 (M2a) (9, 10), immune complexes in combination with IL-1β or LPS (M2b) (10, 16), the anti-inflammatory cytokines, IL-10 and TGFβ, or glucocorticoids (M2c) (8, 17), IL-6, LIF, and MCF (M2d) (18). M1 cells display effector, proinflammatory, and Th1-oriented immunostimulatory properties, representing an important source of reactive oxygen and nitrogen intermediates and of proinflammatory Th-1-priming cytokines, mediate antimicrobial defense, tissue destruction, and antitumor resistance (3, 17). Conversely, M2 cells are oriented to Th2-type immunoregulation and resolution of inflammation, exhibit tissue remodeling and repair functions, promote wound healing, angiogenesis, resistance to parasites, and tumor growth through the production of anti-inflammatory cytokines, ECM components, remodeling and proangiogenic factors (1, 8–10, 19). Mf have a remarkable degree of functional plasticity, as exemplified by their ability to rapidly and reversibly shift between different activation states *in vitro* in response to changes in the activating stimulus, overriding the initial M1/M2-polarization (6, 9, 10, 13, 15, 20, 21). Imbalance of M1/M2 polarization or repolarization of resident Mf is often associated with various diseases, and mixed M1/M2 phenotypes have been described in many pathological situations, such as cancer, inflammatory and autoimmune disorders, and chronic infections (8, 9, 22, 23).

The factors regulating Mf polarization are the focus of intense investigation. A large body of evidence indicates that Mf polarization state is not only determined by the type of activating stimulus but also depends on the local tissue environment in which they differentiate (24, 25). A common hallmark of the pathologic microenvironment is represented by hypoxia, a condition of low partial oxygen pressure (pO_2_, 0–20 mm Hg, hypoxia) which arises as a result of disorganized or dysfunctional vascular network and diminished O_2_ supply (5, 26–29). Mf development from Mn precursors recruited to tumors and injured/inflamed tissues occurs in the setting of low pO_2_, and adaptation to the local hypoxic environment is critical for Mf to fulfill their functions at these sites [reviewed in Ref. (5, 7, 24, 26, 30)]. The role of hypoxia in Mf polarization is only beginning to emerge (30), and most information have been obtained from studies in rodent tumor models (31–33). Relevant interspecies variability has been highlighted with regard to the expression of Mf polarization markers and activation programs, cautioning against direct mouse-to-human extrapolation (19, 34–37). Understanding how the hypoxic environment affects human Mf polarization may have important implications for Mf therapeutic reprogramming in chronic inflammatory diseases and tumors.

In this study, we investigated the impact of a hypoxic environment reflecting that occurring in diseased tissues on the polarization of human Mn-derived Mf. We present data showing that development under hypoxic conditions (1% O_2_) skews Mf polarization toward a M2 phenotype and characterize a previously unrecognized role for the triggering receptor expressed on myeloid cells (TREM)-1, a member of the Ig superfamily of immunoregulatory receptors (38, 39) that we have recently identified as a new hypoxia-inducible gene in Mn-derived DCs (40–42), as a critical determinant of hypoxic M2-to-M1 reprogramming. Molecules associated with M1 polarization are being actively sought as potential diagnostic tools and therapeutic targets in chronic inflammatory conditions, including oligoarticular juvenile idiopatic arthritis (OJIA), the most common pediatric chronic rheumatic disease characterized by extensive and persistent synovial Mn cell infiltration and hypoxia (5, 43). We provide evidence that Mf enriched in the synovial fluid (SF) from active joints of OJIA patients exhibit high TREM-1 expression associated with predominant polarization toward the M1 phenotype and that OJIA-SFs exert M1 repolarizing effects on *in vitro*-generated hypoxic M2 cells partially attenuated by TREM-1 blocking with the specific synthetic peptide inhibitor, LP17 (44). Furthermore, we demonstrate the presence in OJIA-SF of high levels of the DAMP molecule, high-mobility group box 1 (HMGB1), a nuclear DNA-binding protein recently reported to be a potential natural ligand of TREM-1 (45, 46).

## Materials and Methods

### Mf Differentiation, Polarization, and Culture

Peripheral blood mononuclear cells were isolated by density gradient centrifugation over a Ficoll cushion (Histopaque 1077; Sigma-Aldrich, Milano, Italy) from platelet apheresis of healthy volunteers (both male and female in a similar proportion) obtained by the Blood Transfusion Center of the Gaslini Institute in Genova, Italy, according to a protocol approved by the Gaslini’s Ethics Committee and in adherence with the Declaration of Helsinki. Written informed consent was obtained from all subjects enrolled in the study. Mn were purified by MACS immunomagnetic positive selection using the human CD14-microbeads isolation kit (Miltenyi Biotec, Bologna, Italy), resulting in preparations with a purity of ≥95%, as determined by flow cytometry upon staining with anti-CD14 mAb. To generate Mf (hereafter referred as M0), primary Mn were cultured for 6 days in six-well culture plates (BD Falcon, Milano, Italy) with RPMI 1640 (Euroclone, Milano, Italy) supplemented with 10% heat-inactivated FCS (HyClone, Thermo Scientific, Milano, Italy) in the presence of 100 ng/mL of the maturation factor, hM-CSF premium grade (Miltenyi Biotec), under normoxic (20% O_2_) or hypoxic (1% O_2_,) conditions at a concentration of 8 × 10^5^ cells/mL. Half medium was replaced at day 3 of culture. Mf differentiation was confirmed by positivity for the pan-Mf marker CD68 (47), which ranged between 97 and 100% among different donors. Mf polarization was obtained by removing conditioned medium (CM) and then culturing M0 Mf for additional 24 h with fresh medium supplemented with 5% FCS and containing 100 ng/mL Ultra-Pure LPS from Escherichia coli O111:B4 (Sigma-Aldrich) (for M1 polarization) or 20 ng/mL hIL-4 (Miltenyi Biotec) (for M2 polarization), as previously described (48, 49), under normoxic or hypoxic conditions. M0 Mf cultured with fresh medium without polarization factors were used as baseline control. Polarization was confirmed by morphological features, assessed under a phase-contrast microscope (Olympus CKX41 equipped with an Ultra20 Olympus Photocamera for image capturing, Olympus Italia Srl, Milano, Italy): M1 cells appeared as adherent cells with a classical fried egg morphology, whereas M2 cells as adherent and stretched, “spindle-like” cells. Hypoxic conditions were obtained by cell incubation and handling in a sealed anaerobic workstation incubator (Ruskinn, INVIVO_2_ 400, CARLI Biotec, Roma, Italy), incorporating a gas mixing system (Ruskinn Gas Mixer Q) and flushed with a mixture of 1% O_2_, 5% CO_2_, and 94% N_2_. Medium was allowed to equilibrate in the hypoxic incubator for at least 2 h before use. Mf from different donors were polarized in independent experiments. In some experiments, hypoxic M2-polarized Mf were cultured in twelve-well flat-bottom culture plates (Corning Life Sciences, Milano, Italy) and incubated for 24 h with SF from OJIA patients (see below) diluted 1:2 with fresh medium in the presence or absence of 100 ng/mL of the TREM-1-specific 17-mer peptide, LP17, or a scrambled control peptide (Pepscan, Lelystad, The Netherlands), chemically synthesized as described by Gibot et al. (44).

### SF and SF Mononuclear Cell (SFMC) Isolation

Synovial fluid samples were obtained at the time of therapeutic knee arthrocentesis from six children affected by active OJIA with synovial effusion, according to the International League of Associations for Rheumatology criteria (43) and collected into sodium-heparin tubes under vacuum. pO_2_ levels in SF samples were monitored to confirm hypoxic conditions, and specimens were handled in the anaerobic incubator to prevent cell reoxygenation, as detailed (40, 50, 51). Paired peripheral blood (PB) samples were collected in EDTA tubes on the occasion of routine venepuncture. PB from five age-matched control subjects (three females and two males) undergoing venepuncture for minor orthopedic procedures was used as a control. Cell-free SF and plasma were obtained by centrifugation of the specimens and stored at −80°C until assayed. SFMCs were isolated over a Ficoll cushion, as previously described (40, 50, 51). Informed written consent was obtained in accordance with the guidelines of the Gaslini’s Ethical Committee. Clinical and laboratory characteristics of patients at the time of sampling are reported in Table 1.

**Table 1:** Clinical and laboratory features of OJIA patients at the time of sampling.^a^

Disease subtype	Age at sampling (years)	Disease duration (years)	Female/male	ESR (mm/h)	CRP (mg/dL)	RF^**+**^ (%)	ANA	Previous treatment
Persistent oligoarticular (*n* = 3)	11.9^b^ (9.8–14)	4.7 (1.9–8.1)	3/0	18 (1–40)	0.7 (0.46–1.2)	0	−(1 patient) + (2 patients)	Nil (3 patients)
Extended oligoarticular (*n* = 3)	15.2 (8.7–20.8)	12. 4 (4.5–18)	2/1	53 (17–88)	1.8 (0.46–2.5)	0	+(3 patients)	MTX(2 patients) MTX + Etanercept (1 patient)

*^a^Disease activity was defined by the presence of joint swelling or limitation of movement with either pain on movement or tenderness*.

*^b^Results are expressed as means (ranges in parentheses)*.

*ESR, erithrocyte sedimentation rate; CRP, C-reactive protein; RF, rheumatoid factor antibody; ANA, antinuclear antibody; MTX, methotrexate; Nil, no treatment*.

### Flow Cytometry

Flow cytometry was performed as described (40). Mf were resuspended with FACS buffer (PBS supplemented with 0.2% BSA, 0.01% NaN_3_) and stained with PE-conjugated mouse anti-human mAbs against CD14, CD80, CCR7, and TREM-1 (clone 193015, obtained from BioLegend, Milano, Italy), CD68 (obtained from Dako, Milano), CD206 (obtained from Miltenyi Biotec), CD86, HLA-DR, CD36 (BD-Pharmingen, Milano, Italy), and isotype-matched IgG (obtained from BioLegend) for 30 min at 4°C, after preincubation with rabbit IgG (obtained from Sigma) to block non-specific sites. Four-color flow cytometric analysis was carried out to analyze SFMCs using the following Abs: anti-CD68-FITC (obtained from Dako), anti-CD80-PE/Cy7, anti-CD206-APC, and anti-TREM-1-PE Abs (obtained from Biolegend). Fluorescence was quantitated on a FACSCalibur flow cytometer equipped with CellQuest software (BD-Biosciences). Cells were gated according to their light-scatter properties to exclude cell debris.

### Quantitative Real-time RT-PCR

Total RNA was purified from M1 and M2-polarized Mf using Trizol (Life Technologies, Monza, Italy) and controlled for integrity with an Agilent Bioanalyzer 2100 (Agilent Technologies, Waldbroon, Germany). RNA was quantified by NanoDrop spectrophotometry (NanoDrop Technologies, Wilmington, NC, USA) and reverse-transcribed into cDNA on a GeneAmp PCR System 2700 thermal cycler (Applied Biosystems, Milano, Italy) using the SuperScript Double-Stranded cDNA synthesis kit (Life Technologies). qRT-PCR was performed on a 7500 Real Time PCR System (Applied Biosystems) in triplicate for each target transcript using SYBRGreen PCR Master Mix (Life Technologies) and sense/antisense oligonucleotide primers synthesized by TIBMolbiol (Genova, Italy) or from Qiagen (Milano, Italy) (ribosomal protein S19, RSP19), as detailed (52). Expression data were normalized on the values obtained in parallel for three reference genes (*actin-related protein 2/3 complex 1B, ARPC1B; lysosomal-associated multispanning membrane protein-5, LAPTM5; RPS19*), using the Bestkeeper software, and relative expression values were calculated using Q-gene software, as detailed (52).

### ELISA

Soluble (s)TREM-1 was quantified in cell-free Mf supernatants by human TREM-1 DuoSet (R&D Systems). Secreted IL-12, TNFα, IL-1β, CXCL8, IL-6, and IL-10 (Peprotech, Milano), osteopontin (OPN), CCL18, CCL24 and TGFβ1 (R&D Systems, Space Import Export, Milano, Italy) content in Mf CM was also measured by specific ELISA. HMGB1 levels were measured in SF and plasma from OJIA patients and age-matched controls using HMGB1 ELISA kit II (IBL International, Hamburg, Germany); the lower limit of detection was 0.3 ng/mL. Optical density was determined using a Spectrafluor Plus plate reader from TECAN (Milano, Italy). All assays were done in duplicate.

### TREM-1 Crosslinking

Twelve-well flat-bottom culture plates were precoated with 10 µg/mL of agonist anti-TREM-1 mAb (clone 193015, R&D Systems catalog no. MAB1278) or control IgG1 (R&D Systems) diluted in PBS overnight at 37°C. Eight ×10^5^ M1 or M2-polarized Mf/mL of fresh RPMI 1640 w/o cytokines were seeded in each well, briefly spun at 130 *g* to engage TREM-1, and then incubated for 24 h under hypoxic conditions. Culture supernatants were then harvested by centrifugation and tested for cytokine/chemokine content by ELISA, and Mf were analyzed phenotypically.

### Ag Uptake Assay

The 1 × 10^6^ cells were incubated with FITC-labeled dextran beads (1 mg/mL; Sigma) in culture medium under normoxic or hypoxic conditions. After an incubation period of 30 or 60 min at 4°C and 37°C, for background and active uptake values, respectively, cells were extensively washed with ice-cold PBS, and fluorescence was measured on a FACSCalibur.

### Statistical Analysis

Statistical analyses were performed using the GraphPad Prism 5 software (GraphPad Software, La Jolla, CA, USA). Data are expressed as the mean ± SEM of at least three independent experiments, unless differently specified. Statistical significance was evaluated by two-tailed paired Student’s *t*-test, unless differently specified. A *p* value <0.05(*), <0.01(**), or <0.001(***) was considered statistically significant.

## Results

### LPS- and IL-4-Treated Human Mf Exhibit Surface Marker and Secretory Profiles Typical of M1- and M2-Polarized Subsets

M1 and M2 polarization states are defined by specific phenotypic and secretory patterns (17, 53, 54). Human Mf (M0) were generated by culturing primary Mn with the maturation factor, M-CSF, and then polarized toward the M1 and M2 activation states by stimulation with LPS and IL-4, respectively. Surface expression of the Mf differentiation marker, CD68, the prototypical M1 marker, CD80, and the M2 marker, CD206, was then analyzed by flow cytometry to confirm polarization (Figures S1A,B in Supplementary Material). Panel A depicts the staining pattern of untreated, LPS, and IL-4-treated Mf from a representative donor and Panel B shows the percentage and mean fluorescence intensity (MFI) of CD80^+^ and CD206^+^ cells in LPS- and IL-4-polarized Mf from five different samples. CD68 was expressed at high levels on M0 cells, confirming Mf differentiation and maintained during polarization with both polarization stimuli. Low surface levels of the T cell costimulatory molecule, CD80, were detectable in unpolarized Mf, associated with high expression of the mannose receptor, CD206 in about 30% of cells. LPS-treated Mf exhibited the typical M1 phenotype, characterized by strong induction of CD80 and downregulation of CD206 compared to unpolarized Mf both in terms of percentage of positive cells and MFI. Conversely, IL-4-treated cells displayed phenotypic features of M2-polarized cells, such as high surface expression of CD206, that was upregulated respect to unstimulated Mf, and low levels of CD80. CD80 and CD206 expression were significantly (*p* < 0.001) decreased and increased, respectively, in IL-4- compared to LPS-treated Mf both in terms of percentage of positive cells and MFI.

The content of cytokines/chemokines associated with M1 or M2 activation states was then measured in Mf supernatants by ELISA, using M0 cells as a control (Figure S1C in Supplementary Material). LPS-treated cells released significantly higher levels of the M1-type cytokines, TNFα (*p* < 0.001), IL-12 (*p* < 0.05), and IL-1β (*p* < 0.05) compared to both untreated and IL-4-treated Mf, which failed to release detectable levels of IL-1β and secreted little or negligible amounts of IL-12 and TNFα. In contrast, unstimulated and IL-4-treated Mf released similar high amounts of the M2 cytokines/chemokines, TGFβ and CCL24, that were significantly decreased (*p* < 0.05) by LPS stimulation, whereas CCL18 secretion was significantly increased (*p* < 0.001) in LPS-treated as compared to untreated Mf and further upregulated (*p* < 0.001) upon stimulation with IL-4, in agreement with previous findings (13).

Collectively, these data confirm M1 and M2 polarization state of LPS- and IL-4-treated Mf.

### The Hypoxic Environment Exerts M2-Polarizing Effects on Mf

**Figure 1:**
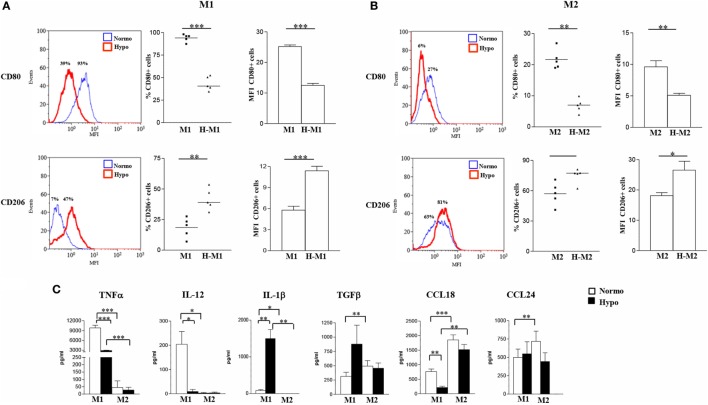
Effects of hypoxia on M1 and M2 polarization markers. M1- and M2-polarized Mf were generated from M-CSF-treated primary Mn stimulated with LPS and IL-4, respectively, under normoxic or hypoxic condition. **(A,B)** Surface marker expression. CD80 and CD206 surface levels were compared by flow cytometry. Histograms depict the results obtained in one representative donor; the blue lines represent the fluorescent profile of normoxic cells, and the red lines represent the fluorescent profile of hypoxic cells stained with the indicated Abs; the percentage of positive cells is indicated. The scatter plots represent the percentage of positive cells from five different donors (dots) with horizontal lines indicating mean values for each group. The bar graphs represent the means of mean fluorescence intensity (MFI) ± SEM of positive cells in five different donors. *p* values of M1 relative to H-M1, M2 relative to H-M2: *p < 0.05; **p < 0.01; ***p < 0.001. **(C)** Cytokine/chemokine secretion. The concentration of the indicated cytokines/chemokines was measured by specific ELISA in the supernatants from normoxic (open bars) and hypoxic (full bars) M1 and M2 cells. Results are expressed as pg/8 × 10^5^ cells/mL and represent the mean ± SEM of five different experiments. *p* values of M1 relative to M2, M1 relative to H-M1, M2 relative to H-M2, H-M1 relative to H-M2: *p < 0.05; **p < 0.01; ***p < 0.001.

To determine the impact of hypoxia on Mf polarization, surface marker expression and cytokine release were compared in M1- and M2-polarized Mf generated under normoxic (20% O_2_) and hypoxic (1% O_2_) (hereafter referred to as H-M1, and H-M2) conditions (Figure 1). The effect of the hypoxic environment on control Mf (hereafter referred to as H-M0) was assessed in parallel (Figure S2 in Supplementary Material). As determined by flow cytometry, Mf generated under hypoxia exhibited different patterns of surface marker expression compared to their normoxic counterparts. Hypoxia led to a significant increase in the percentage (*p* < 0.01) and MFI (*p* < 0.05) of CD206^+^ H-M0 cells, that reached levels comparable to those shown by M2-polarized cells, without substantially affecting CD80 expression (Figure S2A in Supplementary Material). A significant upregulation of CD206 expression was also observed in H-M1 compared to M1 both in terms of percentage of positive cells (*p* < 0.01) and MFI (*p* < 0.001), that reached levels close to those observed in H-M0 cells and was associated with a significant reduction (*p* < 0.001) in the fraction of CD80^+^ cells and the level of expression of the molecule (Figure 1A). Similar to H-M1, H-M2 exhibited significantly decreased (*p* < 0.01) expression of CD80, both in terms of percentage of positive cells and MFI, and increased CD206 surface levels (MFI, *p* < 0.05) respect to M2 cells, although with some variability among individual donors (Figure 1B).

Consistent with phenotypic data, we found profound differences in cytokine/chemokine release between hypoxic and normoxic Mf under both M1- and M2-polarizing conditions (Figure 1C). With regard to the M1-type cytokines, a 70% reduction in the mean amounts of secreted TNFα was measured in the supernatants of H-M1 relative to M1 cells from 5 different donors (from 9,817 ± 803 to 2,897 ± 139 pg/mL; *p* < 0.001), and IL-12 secretion was almost completely inhibited (from 204 ± 53 to 8.8 ± 8.8 pg/mL). Conversely, IL-1β secretion was increased by ≈18-fold (from 83 ± 27 to 1,497 ± 295 pg/mL; *p* < 0.01) under hypoxia. Release of M2-type cytokines was also differentially modulated following M1 polarization under hypoxia. An increase of ≈2.8-fold in the amounts of secreted TGFβ (from 314 ± 7 to 877 ± 332 pg/mL) was measured reaching levels higher, although not significantly, than those released by M2 cells, whereas secreted CCL18 levels were reduced by about 72% (from 761 ± 87 to 210 ± 45 pg/mL; *p* < 0.01) and CCL24 production was not affected (from 500 ± 113 to 548 ± 165 pg/mL). In M2-polarized Mf, hypoxia was effective at inhibiting TNFα (from 45 ± 45 to 27 ± 19 pg/mL), CCL18 (from 1,851 ± 174 to 1,515 ± 179 pg/mL), and CCL24 (from 718 ± 138 to 443 ± 119 pg/mL) release (by a mean of about 50, 20, and 40%, respectively), even if differences did not reach statistical significance given the high variability among different samples, while it did not affect secretion of IL-12 (from 2.4 ± 2.4 to 3.2 ± 3.2 pg/mL), IL-1β (0.0 ± 0.0 pg/mL), and TGFβ (from 493 ± 97 to 456 ± 92 pg/mL). Secretory changes were also induced upon M0 differentiation under hypoxic conditions. Hypoxia significantly (*p* < 0.05) inhibited IL-12 (from 32 ± 9 to 8 ± 7 pg/mL), CCL18 (from 109 ± 28 to 39 ± 21), and CCL24 (from 695 ± 74 to 396 ± 63) secretion (by a mean of about 75, 64, and 43%, respectively) in three different donors, whereas it increased by about 1.5-fold TGFβ release (from 407 ± 149 to 610 ± 185) (Figure S2B in Supplementary Material), without affecting IL-1β and TNFα (data not shown).

We conclude that the hypoxic environment hinders Mf polarization toward the classically activated phenotype and promotes the acquisition of some phenotypic and secretory features typical of alternative activation.

### Hypoxia Triggers Several Phenotypic, Secretory, and Functional Changes in Polarized Mf

**Figure 2:**
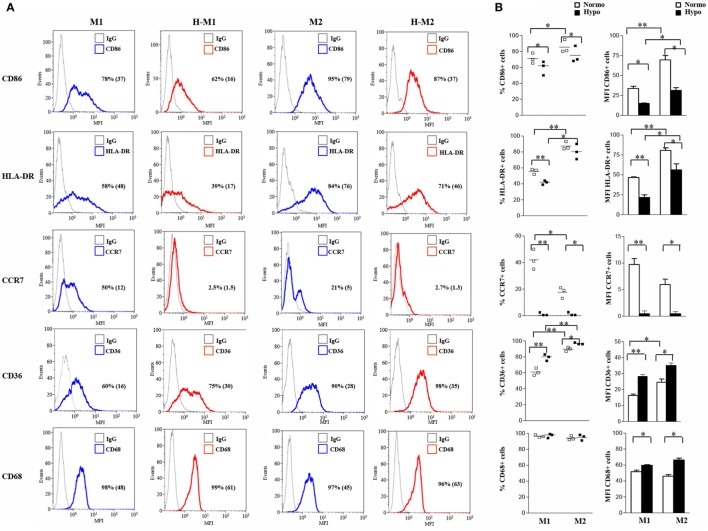
Differential regulation by hypoxia of phenotypic marker expression in M1/M2-polarized Mf. M1/M2 and H-M1/H-M2 cells were generated as described in the legend of Figure 1. Cells were stained with PE-conjugated Abs to CD86, CCR7, HLA-DR, CD36, and CD68 or isotype-matched controls and analyzed by flow cytometry. Results are expressed as detailed in the legend of Figures 1A,B. **(A)** Histograms depict the results obtained in one of three independent donors; positive cell percentage and mean fluorescence intensity (MFI) (between brackets) are indicated. **(B)** The scatter plots represent the percentage of positive cells from three different donors (dots) with horizontal lines indicating mean values for each group. The bar graphs represent the means of MFI ± SEM of positive cells in three different donors. *p* values of M1 relative to M2, M1 relative to H-M1, M2 relative to H-M2, H-M1 relative to H-M2: *p < 0.05; **p < 0.01.

To better characterize the effects of hypoxia on Mf polarization, we investigated the relative expression of surface molecules involved in migration, T cell activation, Ag presentation, and scavenging in Mf polarized under normoxic and hypoxic conditions (Figure 2), compared to control M0 (Figure S2A in Supplementary Material). In line with previous observations (22, 49, 53, 54), elevated membrane levels of the CD86 costimulatory receptor, the MHC class II molecule, HLA-DR, and the scavenger receptor, CD36, were constitutively expressed on Mf, although expression tended to be higher in M2- and lower in M1-polarized Mf as compared to the unpolarized counterpart. Conversely, the percentage of Mf expressing the homing chemokine receptor, CCR7, was increased by M1-polarization and decreased by M2-polarization, confirming earlier reports (19, 54). Interestingly, hypoxia significantly decreased (*p* < 0.05) the percentage of CD86^+^ and HLA-DR^+^ cells and/or their expression levels in both M1 and M2 polarized Mf compared to their normoxic counterpart and almost completely inhibited CCR7 expression, while significantly enhancing the percentage of CD36 positive cells and their MFI (*p* < 0.01 in H-M1, *p* < 0.05 in H-M2) as well as CD68 MFI (*p* < 0.05) (Figure 2). Significant downregulation of CCR7 and upregulation of CD36 in response to hypoxia were also observed in uncommitted Mf (cell percentage, *p* < 0.01; MFI, *p* < 0.05) (Figure S2A in Supplementary Material).

**Figure 3:**
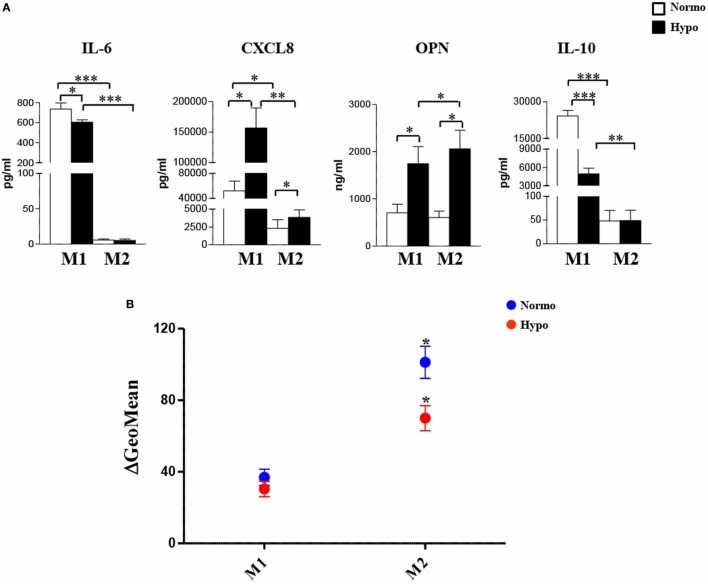
Modulation by hypoxia of cytokine/chemokine secretion and endocytic activity in M1/M2-polarized Mf. M1/M2 and H-M1/H-M2 cells were generated as described in the legend of Figure 2. **(A)** Cytokine/chemokine secretion. Cell-free supernatants were harvested from M1/M2 (open bars) and H-M1/H-M2 (full bars) cultures and assayed for the indicated cytokine/chemokine content by ELISA. Results are expressed as pg/8 × 10^5^ cells/mL (IL-6, CXCL8, IL-10) or ng/8 × 10^5^ cells/mL (OPN) and represent the mean ± SEM of five different experiments *p* value: *p ≤ 0.05; **p ≤ 0.01; ***p < 0.001. **(B)** Endocytic activity. M1/M2 and H-M1/H-M2 cells were incubated for 60 min with FITC-dextran at 4 and 37°C under normoxic and hypoxic conditions, and dextran uptake was determined by flow cytometry. Results are expressed as geometric mean fluorescence intensity (MFI) of cells taking up dextran at 37°C subtracted from that of cells incubated at 4°C (ΔGeoMean). The mean ± SEM of three independent experiments is shown. *p* values of M1 relative to M2 and H-M1 relative to H-M2: *p ≤ 0.05.

We then investigated the release of a set of cytokines/chemokines known to be modulated by hypoxia (55). As depicted in Figure 3A and Figure S2B in Supplementary Material, M1-polarized Mf secreted significantly higher levels of IL-6 (735.6 ± 62 pg/mL; *p* < 0.001), CXCL8 (51.8 ± 15.7 ng/mL; *p* < 0.05), and IL-10 (24,162 ± 2,301 pg/mL; *p* < 0.001) compared to both M2 (IL-6, 5.8 ± 1.5 pg/mL; CXCL8, 2.3 ± 1.2 ng/mL; IL-10, 48 ± 22.1 pg/mL) and unpolarized (IL-6, 8 ± 4 pg/mL; CXCL8, 0.365 ± 0.07 ng/mL; IL-10, 9.7 ± 2 pg/mL) Mf, whereas all Mf subsets secreted comparable high amounts of osteopontin (OPN) (M0, 1,057 ± 252 ng/mL; M1, 707 ± 181 ng/mL; M2, 607 ± 134 ng/mL). Secretion of CXCL8 and OPN was increased by about 3- and 1.7-folds (156.8 ± 32.9 vs. 3.85 ± 1 ng/mL; *p* < 0.05) and 2.5- and 3.4-fold (1,746 ± 361 vs. 2,060 ± 393 ng/mL; *p* < 0.05) in H-M1- and H-M2-cells, respectively, compared to their normoxic counterparts (Figure 3A). OPN secretion levels were also increased although not significantly (1,500 ± 32 ng/mL) in the supernatants of H-M0 compared to M0 cells, whereas CXCL8 release was downregulated by about 70% (111 ± 19 pg/mL, *p* < 0.05) (Figure S2B in Supplementary Material). A 20 and 80% reduction in the amounts of secreted IL-6 (606 ± 23.7 vs. 735 ± 62 pg/mL; *p* < 0.05) and IL-10 (4,940 ± 934 vs. 24,162 ± 2,301 pg/mL; *p* < 0.001), respectively, was measured in the supernatants of H-M1 relative to M1 cells (Figure 3A). Inhibition of IL6 (by 84%; 1.3 ± 1.3 pg/mL) and IL-10 (by 60%; 4 ± 2.3 pg/mL, *p* < 0.01) release was also observed in H-M0 respect to M0 cells (Figure S2B in Supplementary Material), whereas no differences in their secretion were observed between H-M2 and M2 cells (Figure 3A).

Macrophages are important endocytic cells, and polarization is associated with changes in endocytic activity (3, 17). Experiments were thus carried out to investigate whether hypoxia affected endocytic activity of M1- and/or M2-polarized cells, by assessing their ability to take up FITC-labeled dextran. At 4°C, both M1 and M2 cells were unable to capture Ag because of inhibition of cell metabolism, whereas at 37°C, dextran uptake was significant after 30 min and progressively increased until 1 h in both cell subsets (data not shown). As depicted in Figure 3B, M2 cells displayed significantly higher (*p* < 0.0.05) dextran uptake than M1 cells, in line with previous evidence (13, 22). Differentiation under hypoxia caused a 40% decrease in dextran uptake by M2 cells, whereas it only slightly decreased that by M1 cells.

Overall, these data demonstrate that the response pattern of both M1- and M2-polarized Mf is markedly modulated upon generation in a hypoxic environment, resulting in decreased expression of molecules involved in migration, T cell activation, Ag presentation, associated with impaired Ag uptake activity, and in enhanced scavenging receptor expression and proangiogenic cytokines/chemokines production.

### Hypoxia Is an Inducer of TREM-1 Expression in M1- and M2-Polarized Mf

**Figure 4:**
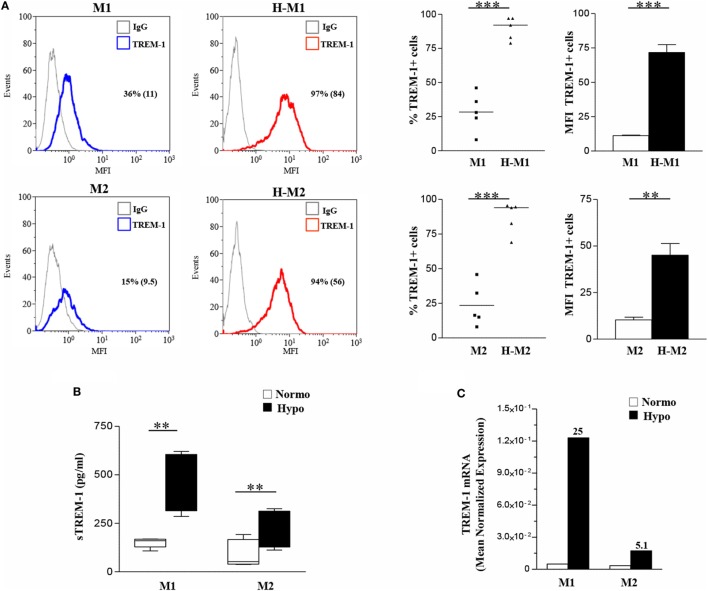
TREM-1 upregulation in H-M1- and H-M2-polarized Mf. M1/M2 and H-M1/H-M2 cells were generated as described in the legend of Figure 2. **(A)** TREM-1 surface expression. Cells were stained with PE-conjugated TREM-1 Ab, and TREM-1 membrane expression was assessed by flow cytometry. Results are expressed as in the legend of Figure 2. Histograms depict the results obtained from one representative experiments of five performed; positive cell percentage and mean fluorescence intensity (MFI) (between brackets) are indicated. Scatter plots represent the percentage of TREM-1-expressing cells from five different donors (dots), with horizontal line representing mean values for each group. The bar graphs represent the MFI ± SEM of TREM-1^+^ cells in five different donors. *p* values of M1 relative to H-M1, M2 relative to H-M2: **p < 0.01; ***p < 0.001. **(B)** sTREM-1 secretion. sTREM-1 concentration in cell-free supernatants was measured by ELISA in the same preparations analyzed in panel A. Results are shown as a box plot and expressed as pg/8 × 10^5^ cells/mL. Boxes contain the values falling between the 25th and 75th percentiles, horizontal lines represent mean values, and whiskers (lines that extend from the boxes) represent the highest and lowest values for each group. *p* values of M1 relative to H-M1 and M2 relative to H-M2: **p < 0.01. **(C)** TREM-1 mRNA expression. TREM-1 transcript levels were quantified by qRT-PCR in total RNA. Data are expressed as mean normalized expression, calculated on the basis of triplicate measurements for each experiment/donor, relative to the values obtained in parallel for three reference genes. Results from a representative of five tested donors are shown. Fold increase values in hypoxic versus normoxic (considered as equal to 1) cells are indicated by a number associated with each bar.

Monocyte-lineage cell activation by hypoxia is associated with specific changes in the expression profile of a cluster of genes coding for immunoregulatory receptors (40, 41, 52). Among them, we have recently identified TREM-1 as a common hypoxia molecular target in both primary Mn and Mn-derived DCs and Langerhans cells and an important regulator of their functions in a hypoxic environment (56). We were interested in investigating whether TREM-1 could affect the polarization of hypoxic Mf. Initial experiments were carried out to assess TREM-1 expression on polarized Mf. M0 Mf were tested in parallel experiments. As measured by flow cytometry, membrane-bound TREM-1 was constitutively expressed at low levels on both unpolarized (Figure S2C in Supplementary Material, *upper panels*) and polarized (Figure 4A) Mf, although M1 polarization induced higher expression, in line with previous results showing LPS stimulatory role on TREM-1 expression (38). A significant increase in the percentage of TREM-1^+^ cells and their MFI was observed in all subtypes upon differentiation under hypoxia, with the extent of induction higher in H-M1 and H-M2 (*p* < 0.01) (Figure 4) than in H-M0 (*p* < 0.05) (Figure S2C in Supplementary Material, *upper panels*). Accordingly, release of the soluble form of TREM-1 (sTREM-1), derived from the shedding of membrane-bound TREM-1 (56, 57), was significantly increased in the supernatants of H-M1 and H-M2 (*p* < 0.01) (Figure 4B), and at a lower extent in those of M0 cells (*p* < 0.05) (Figure S2C in Supplementary Material, *lower panel*), compared to their normoxic counterparts (ranging from 150 ± 11 to 452 ± 66 pg/mL in M1, from 93 ± 31 to 210 ± 43 in M2, and from 6 ± 3.7 to 47 ± 12 in M0 in different donors). Consistent with the protein expression data, marked upregulation of TREM-1 transcript levels were detected by qRT-PCR in hypoxic relative to normoxic polarized cells (Figure 4C), with the extent of increase ranging from 10- to 25-fold and from 4- to 10-fold in H-M1 and H-M2 from five tested donors, respectively.

These findings demonstrate that hypoxia is a critical determinant of TREM-1 expression in Mf and that its stimulatory effects are enhanced following polarization.

### TREM-1 Engagement on Hypoxic Mf Imparts a Proinflammatory M1-Skewed Phenotype to Mf

**Figure 5:**
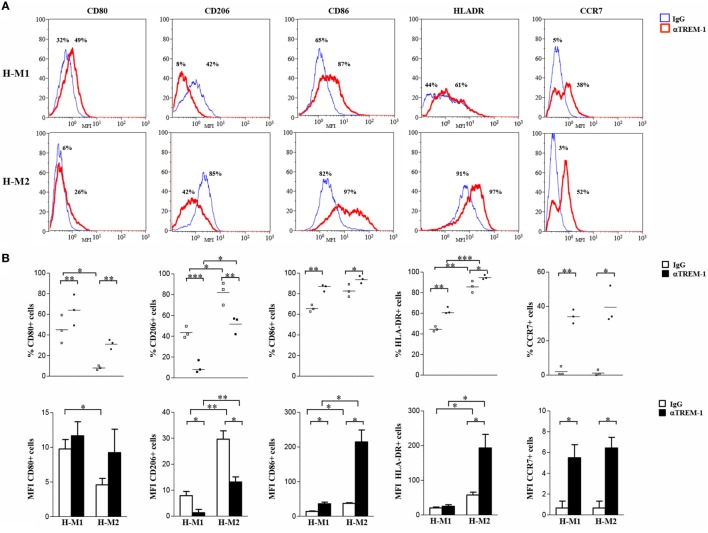
Modulation of H-M1 and H-M2 cell phenotype by TREM-1-cross-linking. H-M1 and H-M2 cells generated as described in the legend of Figure 2 were seeded onto plates pre-coated with agonist anti-TREM-1 mAb or control IgG and cultured for additional 24 h under hypoxic conditions. Cells were then harvested, and surface expression of the indicated markers was determined by flow cytometry. **(A)** Histograms depict the results obtained from a representative of three tested donors. The blue and the red lines represent, respectively, the fluorescent profile of IgG- and TREM-1-triggered cells stained with the indicated Abs; positive cell percentages are indicated. **(B)** The scatter plots represent the percentage of positive cells from three different donors (dots) with horizontal lines indicating mean values for each group. The bar graphs represent the means of MFI ± SEM of positive cells in three different donors. *p* value of TREM-1- relative to IgG-triggered H-M1/H-M2 cells: *p ≤ 0.05; **p ≤ 0.01.

Crosslinking experiments were then carried out to investigate the impact of TREM-1 activation on Mf polarization under hypoxia. To this aim, H-M1 and H-M2 cells were cultured for additional 24 h in plates pre-coated with a specific anti-TREM-1 agonist mAb and then phenotypically and functionally characterized. As determined by flow cytometry (Figure 5), the percentage of cells expressing CD80 (*p* < 0.01), CD86 (*p* < 0.05), CCR7 (*p* < 0.05), and HLA-DR (*p* < 0.05) was significantly increased in response to TREM-1 triggering, as compared to cells crosslinked with an irrelevant isotype-matched control mAb, whereas surface levels of CD206 were significantly (*p* < 0.01) downregulated, in both subsets.

**Figure 6:**
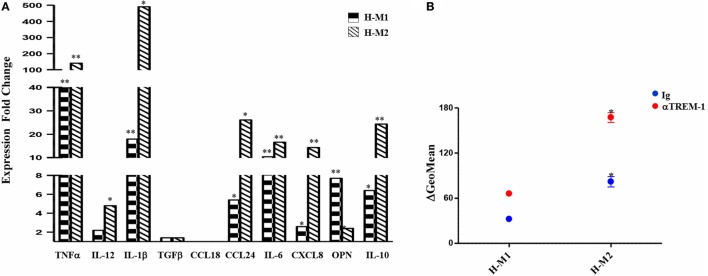
Modulation of H-M1 and H-M2 secretory features and endocytic activity by TREM-1-cross-linking. H-M1 and H-M2 cells were crosslinked with the anti-TREM-1 Ab as described in the legend of Figure 5. **(A)** Cytokine/chemokine secretion. Supernatants were assayed for the indicated cytokines/chemokines content by specific ELISA. Data shown are expressed as fold changes in TREM-1- relative to IgG-crosslinked cells (arbitrarily defined as equal to 1) and represent the mean of five independent experiments. **(B)** Endocytic activity. Ig- and TREM-1-triggered H-M1/H-M2 cells were incubated with FITC-dextran at 4 and 37°C for 1 h under normoxic and hypoxic conditions, respectively, and then analyzed by flow cytometry. Results are expressed as detailed in the legend of Figure 4B and represent the mean ± SEM of three independent experiments. *p* value of TREM-1-triggered H-M2 relative to TREM-1-triggered H-M1 and IgG-triggered H-M2 relative to IgG-triggered H-M1 cells: *p ≤ 0.05.

Supernatants from TREM-1-triggered H-M1 and H-M2 cells were collected and analyzed for cytokine and chemokine content by ELISA. As depicted in Figure 6A, TREM-1 engagement resulted in a significant and consistent increase in the secreted amounts of the M1 cytokines, TNFα and IL-1β, and in *de novo* release of low levels of IL-12 by both subsets. Among M2 cytokines, TREM-1-triggered H-M1- and H-M2 produced increase amounts of CCL24, whereas no effect was observed on the production of TGFβ and CCL18 regardless of Mf polarizing conditions. TREM-1 crosslinking also led to increased secretion of IL-6, CXCL8, OPN, and IL-10 by both H-M1 and H-M2 cells, as compared to the IgG-triggered counterparts. Interestingly, the stimulatory effect of TREM-1 on all tested cytokines/chemokines, with the exception of OPN, was higher in H-M2- than H-M1-polarized Mf, indicating that the extent of Mf responsiveness to TREM-1 activation depends on the type of pre-stimulation. No substantial differences in marker expression and cytokine secretion were observed upon macrophage crosslinking with an isotype-matched anti-HLA-1 mAb as compared to control IgG, confirming that Mf activation by anti-TREM-1 was specific and not the result of Fc receptor ligation (data not shown).

We then investigated whether Mf endocytic ability was modulated upon TREM-1 triggering by comparing the ability of TREM-1- and control IgG-triggered H-M1 and H-M2 cells to take up dextran. As shown in Figure 6B, both H-M1 and H-M2 cells responded to TREM-1 activation by a twofold increase of dextran uptake, which reached levels higher than those observed in the normoxic counterparts.

Overall, these data suggest that TREM-1 triggering reverses the M2-polarizing effect of hypoxia imparting a M1-skewed proinflammatory phenotype to Mf.

### Mf Infiltrating the Hypoxic SF of OJIA Patients Express TREM-1 and Are Polarized toward a M1 Phenotype

**Figure 7:**
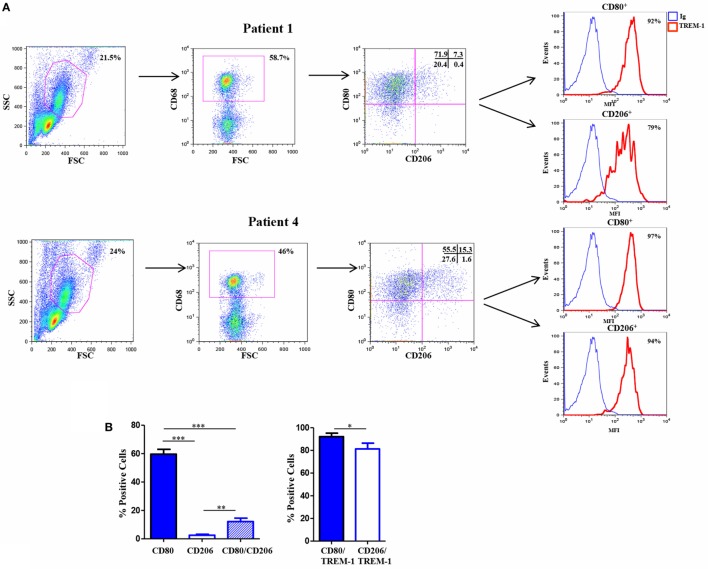
Triggering receptor expressed on myeloid cell (TREM)-1 expression *in vivo* in Mf from hypoxic oligoarticular juvenile idiopatic arthritis synovial fluid (OJIA-SF). SF mononuclear cells (SFMCs) were purified from children affected by OJIA, stained with anti-CD68-FITC, anti-CD80-PE/Cy7, anti-CD206-APC, and anti-TREM-1-PE mAbs, and analyzed by multicolor flow cytometry on a FACScan. **(A)** Results from two representative of six OJIA patients are shown as flow cytometry plots. Myeloid cell populations were electronically gated according to their forward/side scatter properties. Gated cells were analyzed for CD68 positivity, and CD68^+^ cells were then examined for CD80 and CD206 expression. Non-specific staining was corrected using isotype-matched Abs. The percentage of single and double-positive cells within the CD68-gated population is indicated. Upper left quadrants: CD80^+^/CD206 cells (*M1 phenotype*); lower right quadrants: CD80CD206^+^ cells (*M2 phenotype*). Upper right quadrants: CD80^+^/CD206^+^ cells (*mixed M1/M2 phenotype*); lower left quadrants: control IgGs. Coexpression of TREM-1 in CD80^+^ and CD206^+^ cells was then evaluated. Red histograms represent the fluorescent profile of TREM-1-expressing cells, whereas blue histograms represent the fluorescent profile of cells stained with the isotype-matched control Ab. The percentage of TREM-1^+^ cells in the total CD80^+^ and CD206^+^ populations is indicated **(B)** Percentages of CD80 and CD206 single and double-positive cells within the CD68-gated population and percentages of TREM-1^+^ cells in the total CD80^+^ and CD206^+^ subsets are presented as bar graphs. Results are the mean ± SEM of six independent experiments. *p ≤ 0.05; **p ≤ 0.01; ***p ≤ 0.001.

Triggering receptor expressed on myeloid cell 1 has been implicated in the pathogenesis of several infectious and non-infectious chronic inflammatory disorders, including rheumatic diseases (58–61), which are characterized by hypoxia and high Mn cell infiltration (5). Experiments were carried out to assess expression of TREM-1 and Mf phenotype in the SF of patients affected by the pediatric chronic rheumatic condition, OJIA (OJIA-SF). To this aim, SFMCs were isolated from the active knee joints of six OJIA patients and subjected to four-color flow cytometric analysis with mAbs to CD68, CD80, CD206, and TREM-1 (Figure 7). A large subset of SFMCs expressed CD68, confirming Mf enrichment in OJIA-SF. An elevated percentage of CD68^+^ cells, representing ≈60% of the total CD68-gated population in the six patients analyzed, expressed the CD80 marker (Figure 7A, *upper left quadrants;* Figure 7B), whereas only the 2.5% of CD68^+^ cells expressed the CD206 marker (Figure 7A, *lower right quadrants;* Figure 7B), suggesting predominant polarization toward a M1 proinflammatory phenotype. A subset of CD68^+^ cells, that represented ≈12% of the total CD68-gated cells exhibited a mixed M1/M2-type phenotype coexpressing the CD80 and CD206 markers (Figure 7A, *upper right quadrants;* Figure 7B). Interestingly, high TREM-1 expression was detected on the majority of CD80^+^ (about 92%) and CD206^+^ (about 81%) Mf (Figures 7A,B). These findings demonstrate that Mf generated from Mn recruited to the OJIA-SF express high levels of TREM-1 and are mostly polarized toward a M1 phenotype.

**Figure 8:**
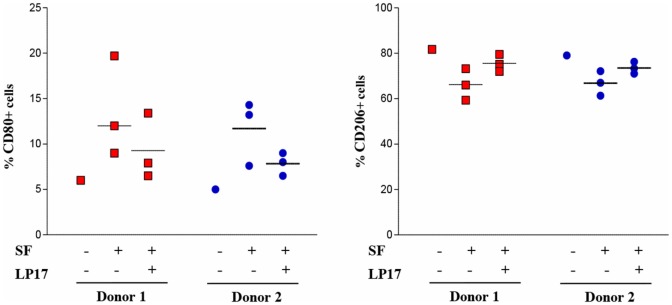
Modulation of CD80 and CD206 polarization markers by oligoarticular juvenile idiopatic arthritis synovial fluid (OJIA-SF) and LP17 peptide in H-M2 cells. H-M2 cells generated from two different donors were cultured for 24 h under hypoxic conditions with SFs from the OJIA patients analyzed in Figure 7 (three SFs for each H-M2 preparation) in the presence or absence of the TREM-1-specific peptide, LP17 (100 ng/mL). Surface levels of CD80 and CD206 were then determined by flow cytometry. Results are expressed as in the legend of Figure 2B. Scatter plots represent the percentage of positive H-M2 cells untreated or treated with three different SF ± LP17 (dots), with horizontal lines indicating mean values for each group. *p* value of SF + LP17- relative to SF-treated cells (by two-tailed paired Student’s *t*-test): *p ≤ 0.05.

Given the predominant M1 polarization of TREM-1^+^ SF-Mf and the presence of a Mf subset with a mixed M1/M2 phenotype, probably representing M2-to-M1 switching cells, we asked whether SFs from OJIA patients could exert M2-to-M1 repolarizing effects via TREM-1 activation. To address this question, we evaluated the expression of CD80 and CD206 polarization markers on *in vitro* polarized H-M2 cells incubated for 24 h with OJIA-SFs in the presence or absence of the synthetic peptide, LP17, derived from a short highly conserved extracellular region of TREM-1 involved in ligand binding (44). As determined by flow cytometry (Figure 8), the percentage of H-M2 cells expressing CD80 was increased, whereas that of CD206 was decreased, following exposure to SFs as compared to the unexposed counterparts, although variability in the response to different SFs was observed, and LP17 addition to the culture partially attenuated these effects. The control peptide displayed no activity on phenotypic marker expression (not depicted). These data suggest that OJIA-SF can promote some M2-to-M1 repolarization and that this response is mediated at least in part by TREM-1 activation, raising the possibility that putative TREM-1 ligands may be present in OJIA SF.

**Figure 9:**
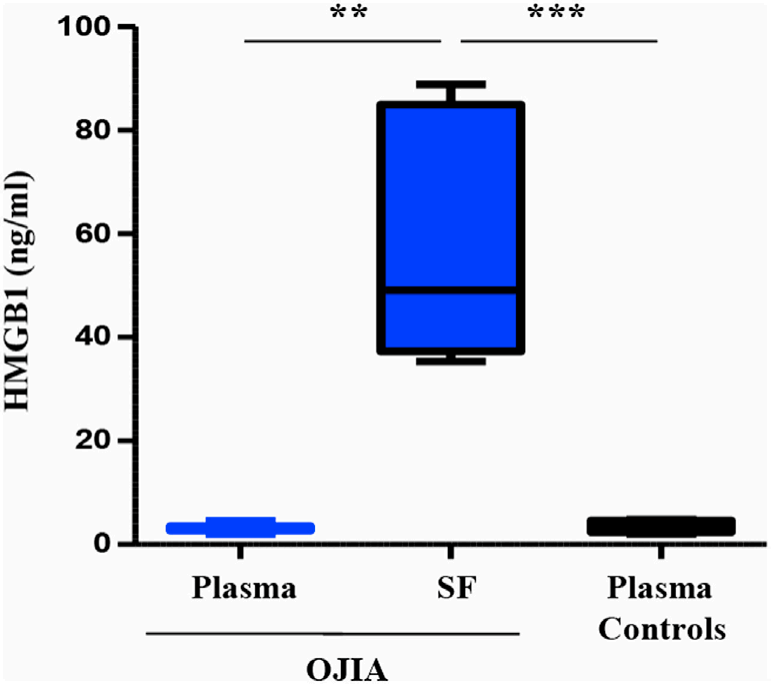
High-mobility group box 1 (HMGB1) release in synovial fluid (SF) and plasma from patients affected by oligoarticular juvenile idiopatic arthritis (OJIA). HMGB1 concentrations were quantified by ELISA in paired plasma and SF from the OJIA patients analyzed in Figure 7 and in plasma from five age-matched control subjects. Individual samples were run in duplicate. Results are expressed as ng/mL Boxes show the values falling between the 25th and 75th percentiles, horizontal lines represent mean values, and whiskers the highest and lowest values for each group. *p* value of SF relative to paired OJIA plasma (by two-tailed paired Student’s *t*-test): **p < 0.01; *p* value of SF relative to plasma controls (by two-tailed unpaired Student’s *t*-test): ***p < 0.001.

Previous reports have suggested a role for TREM-1 in the recognition of DAMPs (45, 56), endogenous molecules released by activated inflammatory and/or damaged/necrotic cells and implicated to the initiation and perpetuation of the inflammatory process in various inflammatory arthritic diseases (62). In particular, the nuclear DNA-binding protein, HMGB1, has been recently identified as a natural ligand of TREM-1 in mice (46). We were, thus, interested in investigating whether HMGB1 was present in the SF from OJIA patients. HMGB1 concentrations were measured by ELISA in paired SF and plasma samples from OJIA patients and in plasma from five age-matched control subjects, used as control. As shown in Figure 9, elevated levels of HMGB1 were detectable in SF from OJIA patients (median 57.3 ± 9.5, range 35–89 ng/mL), that were significantly increased as compared to those present in paired (median 3.1 ± 0.3 ng/mL, *p* < 0.01) and control (median 3.5 ± 0.45 ng/mL, *p* < 0.001) plasma samples. These results suggest that release of the putative TREM-1 ligand, HMGB1, is induced in the inflamed arthritic environment.

## Discussion

Macrophages can be polarized into specialized functional subsets both under physiological (e.g., embryogenesis and pregnancy) and pathological conditions (e.g., cancer, autoimmune disease, chronic inflammation, infection, and tissue repair) (3, 8). Recent studies have highlighted the complexity of *in vivo* Mf polarization, which goes beyond the simple M1–M2 paradigm and represents a continuum of diverse functional states with intermediate or overlapping features (9, 12, 17, 30). Coexistence of cells in different polarization states and unique or mixed M1/M2 phenotypes have been observed in selected preclinical and clinical conditions, and Mf polarization status was shown to change over time throughout the course of the disease (8, 22, 23). Tissue environment has been implicated as the strongest factor in the determination of Mf activation profile (6). In this study, we present novel data demonstrating that a chronic hypoxic environment reflecting that occurring *in vivo* in diseased tissues critically impacts on the polarization of human Mf, counteracting the effects of classically activating stimuli while promoting the acquisition of some phenotypic and secretory features typical of alternative activation. Furthermore, we provide the first evidence of the role of the TREM-1 receptor in reversing the M2 polarizing effects of hypoxia, driving hypoxic Mf reprogramming toward a M1 proinflammatory direction, with implications for OJIA pathogenesis.

Clear differences in the morphology, phenotype, secreted cytokine pattern, transcriptional profile, and endocytic activity between M1- and M2-polarized Mf have been reported (34, 49, 53, 54, 63). In agreement with data in the literature, LPS- and IL-4-stimulated human Mf displayed the M1 (CD68^+^/CD80^high^/CD206^/low^) and the M2 (CD68^+^/CD80^/low^/CD206^high^), phenotype, respectively, as compared to unstimulated Mf that displayed the M0 phenotype (CD68^+^/CD80^/low^/CD206^low^). Increased surface expression of CCR7, previously associated with M1 polarization (19, 49, 54), was detectable in LPS- respect to IL-4-treated Mf, which conversely expressed higher amounts of the M2 marker, CD36, and of the CD86 and HLA-DR molecules. CD86 and MHC-class II molecules were reported to be only minimally expressed in IL-4-polarized Mf in mice (10), reinforcing the notion that mouse and human Mf differ in the expression levels of some M1–M2 polarization markers (12, 13, 19, 34, 35, 37, 54). Given their role in the balance between Th1 and Th2 development through CD28 engagement on T cells (64), the observation that CD80 and CD86 expression levels are higher in human M1- and M2-polarized Mf, respectively, confirms the respective Th1- and Th2-supporting properties of the two subsets (9, 10). Phenotypic M1 and M2 polarization was paralleled by secretion of a distinct set of cytokines/chemokines, with high amounts of the proinflammatory Th1-priming cytokines, IL-12, TNFα, IL-1β, and IL-6 released by LPS-treated Mf but not by IL-4-treated Mf, that in contrast exhibited increased production of the immunosuppressive cytokine, TGFβ, and the chemokines, CCL18 and CCL24, in agreement with earlier reports (17, 19, 54). Interestingly, higher levels of the anti-inflammatory cytokine, IL-10, were released by M1 than M2-polarized Mf. IL-10 has long been considered as a M2 marker based on studies in mice (9, 10, 17). Conversely our data demonstrate that human M1-polarized Mf are important IL-10 producers, corroborating recent findings (13, 54, 63) and highlighting relevant interspecies variability also with regard to M1–M2 secreted cytokines. The observation that uncommitted M0 Mf produced little or negligible TNFα, IL-1β, IL-6, and IL-10 as well as increased TGFβ and CCL24 and expressed higher CD206 and CD36 than LPS-treated Mf without reaching the levels shown in IL-4-polarized cells suggest that M-CSF-driven Mf maturation induces a moderate M2-oriented activation state. However, the presence of some M1-like features, such as expression of CCR7 and production of IL-12, indicate the lack of complete M2 polarization, that was induced upon treatment with IL-4 and reversed by LPS, supporting previous findings by others (13, 14, 19, 49, 53, 54). In addition to differences in surface marker expression and cytokine production, M1-and M2-polarized Mf differed with regard to endocytic capacity, as shown by a significantly higher (*p* < 0.0.05) dextran uptake by IL-4- compared to LPS-treated cells, in agreement with previous reports (20, 22).

Macrophages response to M1- and M2-polarizing stimuli was markedly modulated upon generation under a hypoxic environment. LPS-treated hypoxic Mf exhibited significantly lower surface levels of the M1 markers, CD80 and CCR7, and concomitant significant increase in the expression of the M2 markers, CD206 and CD36, compared to their normoxic counterparts, thus displaying a phenotype similar to that of IL-4-treated Mf. Accordingly, cytokines/chemokines produced by hypoxic LPS-polarized Mf switched from the M1- to the M2-type. Secretion of IL-12, TNFα, IL-6, and IL-10 was, in fact, significantly reduced whereas that of TGFβ and the proangiogenic mediators, CXCL8 and OPN, was increased under hypoxia, although statistically significant differences were evident only for CXCL8 and OPN. Similarly to LPS-polarized Mf, unpolarized and IL-4-polarized hypoxic Mf exhibited significant upregulation of CD206 and CD36 associated with significant downregulation of CCR7, as well as increased production of OPN, compared to cells differentiated under normoxia, confirming the M2-polarizing effects of hypoxia. Significant downregulation of CD80 and increase of CXCL8 were also observed in IL-4-polarized hypoxic Mf, whereas decreased secretion of IL-12, IL-6, and IL-10 and increased release of TGFβ were induced by hypoxia in M0. These results point to a role of the hypoxic environment as a direct trigger of human Mf polarization toward the M2 activation state, reinforcing the effects of M2-polarizing stimuli and re-educating M1-polarized macrophages toward alternative activation by counteracting the effects of classical activation stimuli. These findings confirm and extend recent reports in TAMs (31, 32, 65, 66), although divergent results have also been obtained (33). Reversal of Mf proinflammatory responses to LPS was previously shown to occur upon stimulation with immunocomplexes (10, 16), and we extend this observation to hypoxia, suggesting that LPS can differently affect Mf functions depending on the type of pre- or costimulation. The observation that secretion of the M1 cytokine, IL-1β, was increased and that of the M2 chemokine, CCL18, was decreased in LPS-treated hypoxic Mf suggest their retainment of some properties typical of M1-polarized Mf, thus predicting a mixed M1/M2 polarization state upon development within pathologic hypoxic tissues. Indeed, a mixed M1/M2 phenotype was reported in TAM (8, 67) and adipose tissue Mf (22).

Impairment of Ag uptake ability is an important effect of hypoxia on Mn-derived immature DCs (iDCs) [for a review see Ref (55)]. Our data show that the hypoxic environment reduced the ability of M1 and M2-polarized Mf to take up dextran. Dextran endocytosis can occur via C-type lectin receptors, such as CD206, and we have previously reported the relation between dextran uptake inhibition in iDCs generated under hypoxia and CD206 downregulation (68). Differently from DCs, inhibition of dextran uptake in Mf could not be ascribed to CD206, because it was upregulated by hypoxia, thus implicating other scavenger receptors in mediating endocytic activity impairment in these cells. Downregulation of different surface endocytic scavenger receptors involved in the uptake of lipids and lipoproteins, apoptotic cells, hemoglobin and glycoproteins in human Mn lineage cells in response to hypoxia has been previously reported (52). Inhibition of Ag uptake ability together with the observed decrease in the expression of HLA-DR, CD80, and CD86 molecules, which are involved in Ag presentation and T cell costimulation, suggest that Mf generated at pathologic hypoxic sites are less efficient in triggering T cell responses compared to Mf generated under physiological conditions. Furthermore, the evidence that secretion of IL-12, the main Th1-priming cytokine (69), and CCL18, a specific chemoattractant and activating cytokine for naive T cells and iDCs (70), was inhibited under hypoxia in M1-polarized Mf raises the possibility that their Th1-priming ability is impaired under hypoxic conditions, and further investigations are underway to address this hypothesis. Interestingly, these results confirm and extend our earlier evidence in Mn-derived Langerhans cell showing that hypoxia inhibits their expression of T cell costimulatory molecules and impairs their stimulatory activity on naive T cells (42), but differ from previous findings in Mn-derived DCs that demonstrated CD80 and CD86 molecule overexpression upon differentiation under conditions of reduced oxygenation (41, 68, 71). Taken together, these findings strongly suggest that hypoxia can differentially modulate the T cell stimulatory activity of distinct Mn lineage cell subsets.

Monocyte cell migratory activity is a tightly regulated process mediated by a specific repertoire of chemokine receptors and adhesion molecules, known to be highly sensitive to microenvironmental changes (7). Low pO_2_ was reported to significantly and differentially influence the chemokine receptor profile and the migratory behavior of cells belonging to the Mn lineage. We showed that hypoxia promoted the onset of a migratory phenotype in Mn-derived iDCs by inducing the expression of receptors for both inflammatory and homeostatic chemokines, such as CCR2, CCR3, CCR5, CXCR4, CX3CR1, with consequent increased responsiveness to specific chemoattractants (68, 71). In contrast, downregulation of CC inflammatory chemokine receptors, namely CCR5, CCR2, and CCR1, was a common feature of primary Mn and Mn-derived Mf response to low pO_2_ and an important mechanism regulating their retainment/concentration in hypoxic areas of inflammatory and tumor lesions (7, 52, 72). The demonstration that hypoxia inhibits CCR7 expression on unpolarized Mf a well as under both M1- and M2-polarization conditions is compatible with the hypothesis that reduced tissue oxygenation impairs Mf trafficking *in vivo*.

Myeloid cells integrate signals present in the microenvironment through a defined repertoire of activating and inhibitory surface immunoregulatory receptors, which play a major role in the regulation of their responses in diseased tissues (56, 73). We demonstrated by gene expression profiling the fine regulatory control exerted by hypoxia on the expression of various members of these receptor family in primary Mn and Mn-derived DCs (40, 41, 52, 55). Elucidation of hypoxia effects on immunoregulatory receptor expression and functions in Mf may help to unravel the mechanisms underlying their polarization at pathologic sites and result in the identification of novel ways of therapeutic manipulation. An intriguing finding of this study is the demonstration that hypoxia strongly induces the expression of one of such receptors, TREM-1, in unpolarized Mf and that this effect is further enhanced in M1- and M2-polarized Mf. This molecule was previously reported to be developmentally regulated in Mn-lineage cells, being constitutively expressed at high levels in primary Mn and completely downregulated during Mn differentiation into DCs and Langerhans cells (40, 41). Our results support these findings, showing low TREM-1 expression on M0-, M1-, and M2-polarized Mf generated under normoxia. Noteworthily, TREM-1 mRNA and membrane-bound receptor levels, as well as release of its soluble form detectable in several inflammatory disorders (56), were consistently and significantly increased upon Mf differentiation under chronic hypoxia. This evidence, together with previous observation showing TREM-1 upregulation in primary Mn in response to hypoxia (52, 74) and inducibility in iDCs, mDCs, and Langerhans cells (40–42), is consistent with a role for hypoxia as a trigger of TREM-1 expression, indicating that hypoxic stimulation can overcome TREM-1 developmental downregulation, and highlights the relevance of TREM-1 as a common marker of distinct Mn lineage cell populations in a hypoxic environment.

Triggering receptor expressed on myeloid cell 1 engagement on hypoxic Mf by an agonist mAb reverted the M2-polarizing effects of hypoxia imparting a proinflammatory M1-skewed phenotype to Mf. Features typical of M1 cells, namely expression of CD80 and CCR7 and secretion of IL-12, TNFα, IL-1β, and IL-6, were in fact stimulated upon TREM-1 crosslinking in hypoxic Mf irrespectively of their initially acquired polarization state and were paralleled by downregulation of the M2 marker, CD206. Upregulation of CD86 and HLA-DR expression and enhanced endocytic activity were also prominent features of TREM-1-triggered hypoxic Mf, suggesting restoration of their T cell stimulatory activity. These results provide the first evidence of TREM-1 ability to induce hypoxic macrophage reprogramming from the M2 to the M1 state, supporting and extending previous observations on macrophage dynamic shifting among functional phenotypes *in vitro* upon sequential or concomitant treatment with different stimuli (6, 10, 15, 20, 21, 53). Increased secretion of classical M1-type proinflammatory cytokines together with that of CXCL8, a strong neutrophil chemoattractant (75), OPN, an important mediator of Mn cell and Th1 lymphocyte recruitment (75, 76), and CCL24, a chemotactic factor for eosinophils, basophils, neutrophils, Th2 lymphocytes, and Mf (77), by TREM-1-triggered hypoxic Mf strongly suggest that sustained TREM-1 induction by the hypoxic environment represents an important mechanism of regulation of Mf-mediated inflammatory leukocyte recruitment/accumulation in pathologic tissues. Given CXCL8 and OPN proangiogenic properties (75, 78), TREM-1 contribution to hypoxic Mf-mediated neovascolarization is also highly predictable. These findings corroborate and extend previous observations showing that TREM-1 engagement promotes the proinflammatory and Th1-priming functions of hypoxic DCs and Langerhans cells and their stimulatory activity on T cells (40, 41), emphasizing the relevance of this molecule in the activation of both innate and adaptive immunity at hypoxic sites.

Triggering receptor expressed on myeloid cell 1 has been implicated in the development and perpetuation of a number of non-infectious chronic inflammatory disorders (56), including adult arthritides (58–61). OJIA is the most common chronic pediatric rheumatic disease, characterized by extensive and persistent inflammatory cell recruitment and retention in the synovium of affected joints, which are crucial to disease pathogenesis, and is an important cause of short- and long-term disability due to bone and cartilage destruction (5, 43). Hypoxia occurs in the inflamed rheumatic synovium in the course of the disease and was reported to play a pathogenetic role in OJIA by triggering further leukocyte infiltration and activation (5, 50, 51). We previously documented the presence of elevated amounts of sTREM-1 in the hypoxic synovial effusions of children affected by OJIA and identified TREM-1^+^ DCs as an important component of SF inflammatory infiltrate and a possible source of sTREM-1 (40, 41). Other groups also showed positivity for TREM-1 on myelomonocytic cells infiltrating synovial joints of rheumatoid arthritis patients (58–60). This study expands previous results providing the first evidence of high TREM-1 expression on CD68^+^ Mf enriched in OJIA-SF and demonstrating predominant polarization of TREM^+^ Mf toward the M1 phenotype, characterized by high CD80 and low CD206 expression. These findings support recent observation in obese patients showing positive correlation between TREM-1 and M1 macrophage marker expression (79) and are consistent with the proinflammatory cytokine profile previously detected in the SF of JIA patients (5, 80, 81). Interestingly, we observed coexpression of CD80 and CD206 in a small fraction of Mf, in line with previous findings in circulating Mn from the systemic form of JIA (82), suggesting the presence of a subset of Mf with a mixed M1/M2 phenotype, probably representing M2-to-M1 switching cells.

Taken together with *in vitro* data, the observed correlation between TREM-1 expression and M1 polarization in OJIA-SF suggest that Mf generated from Mn recruited to the arthritic synovium respond to intra-articular hypoxia upregulating TREM-1, which may represent one of the mechanisms mediating their polarization to a M1 proinflammatory state. We hypothesize that, in the early phase of synovial inflammation, infiltrating Mn are induced by the local hypoxic environment to differentiate into M2-polarized Mf, which likely represents a mechanism to prevent immune cell overactivation and collateral inflammatory tissue damage (30). It is conceivable that at this stage TREM-1 expression is induced on synovial Mf in response to intra-articular hypoxia but that its activation does not occur provided that rapid resolution of inflammation ensues. Conversely, if the inflammatory process progresses, TREM-1 is activated by putative DAMP ligands (45, 56) released in the local arthritic environment as a result of chronic inflammation and/or cartilage/bone erosion (83), triggering M1 polarization of both M2-skewed hypoxic Mf and newly recruited Mn and unpolarized Mf, thus contributing to the amplification of OJIA synovitis. Consistent with this hypothesis is the observation that SFs from OJIA patients have M1 repolarizing activity on H-M2 cells *in vitro*, although with a certain degree of variability probably due to their heterogeneity in immune cell composition and resultant soluble mediator content (5, 43), and that these effects are attenuated at least in part by treatment with the synthetic TREM-1-specific peptide, LP17, which was previously reported to function as an inhibitor of TREM-1 activation *in vitro* in human Mn and neutrophils (44, 84, 85) and *in vivo* in various preclinical models of microbial infections and non-infectious inflammatory conditions [for a review see Ref (56).] by competing with the receptor for ligand binding. Furthermore, the finding that high levels of the DAMP molecule, HMGB1 (62), which was recently identified as a potential natural ligand of TREM-1 in mice (46), are present in the SF of OJIA patients [this study and Ref (86).], confirms the release of TREM-1 putative ligands in the inflamed arthritic environment, warranting future in-depth studies of TREM-1 activation in synovial Mf. Interestingly, the same proinflammatory cytokines released by TREM-1-triggered Mf or other inflammatory cells, e.g., TNFα, as well as bacterial products often present in the inflammatory milieu, can upregulate TREM-1 expression (87, 88), indicating the existence of both autocrine and paracrine positive feedback loops sustaining TREM-1 signaling. The extent of synovial Mf proinflammatory activation in affected joints will, thus, be ultimately dictated by the functional interplay among inflammatory mediators released both by macrophages in response to TREM-1 activation and other innate and adaptive immune cell populations recruited to the hypoxic synovium, which synergistically stimulate each other’s production (69), thereby generating a pathologic cycle of inflammation that leads to the chronicity of the disease. Hence, targeting TREM-1 may potentially have therapeutic benefits in OJIA by reducing synovial M1 polarization and local proinflammatory cytokine and chemokine release. TREM-1 blockade has previously been reported to be effective *in vivo* in murine collagen-induced arthritis, an experimental mouse model of RA, attenuating inflammation and improving the clinical course of the disease (58). The therapeutic efficacy of inhibiting Mn lineage cell-derived proinflammatory cytokines has been previously reported in JIA (89).

In conclusion, results from this study highlight the fine regulatory control exerted by the pathologic hypoxic environment on Mf polarization, suggesting that Mf can blend into various “shades” of activation depending on the degree of local oxygenation, that is quite heterogeneous and rapidly fluctuating in diseased tissues (5, 24). Our results also suggest the potential of TREM-1 induction by intra-articular hypoxia on synovial Mf as a mechanism of amplification and perpetuation of the inflammatory process in OJIA with important therapeutic implications.

## Ethics Statement

This study was carried out in accordance with the guidelines of the Gaslini’s Ethical Committee. Written informed consent was obtained from all subjects enrolled in the study in adherence with the Declaration of Helsinki.

## Author Contributions

FR conducted the experiments, collected, assembled, and analyzed the data; SP, DP, and FP provided experimental support and helped in the analysis of data; MaG provided patient specimens and revised the work; provided financial support; AE revised the manuscript; LV, MiG and FN revised the manuscript and provided financial support; MB conceived the study, designed experiments, interpreted the data, and wrote the manuscript.

## Conflict of Interest Statement

The authors declare that the research was conducted in the absence of any commercial or financial relationships that could be construed as a potential conflict of interest.

## Abbreviations

Mf, macrophages; Mn, monocytes; PAMPs/DAMPs, pathogen- or damage-associated molecular patterns; pO_2_, partial oxygen pressure; TREM-1, triggering receptor expressed on myeloid cells 1; OJIA, oligoarticular juvenile idiopatic arthritis; SF, synovial fluid; HMGB1, high-mobility group box 1; SFMCs, SF mononuclear cells; sTREM-1, soluble TREM-1; OPN, osteopontin; MFI, mean fluorescence intensity; iDCs, immature DCs.
