# Supplementary material for: Regulation of Human Macrophage M1–M2 Polarization Balance by Hypoxia and the Triggering Receptor Expressed on Myeloid Cells-1
Source: Front Immunol. 2017 Sep 7;8:1097. doi: 10.3389/fimmu.2017.01097 (PMC5594076; doi:10.3389/fimmu.2017.01097)
Supplement: Supplementary file 1 [file image_1.pdf]

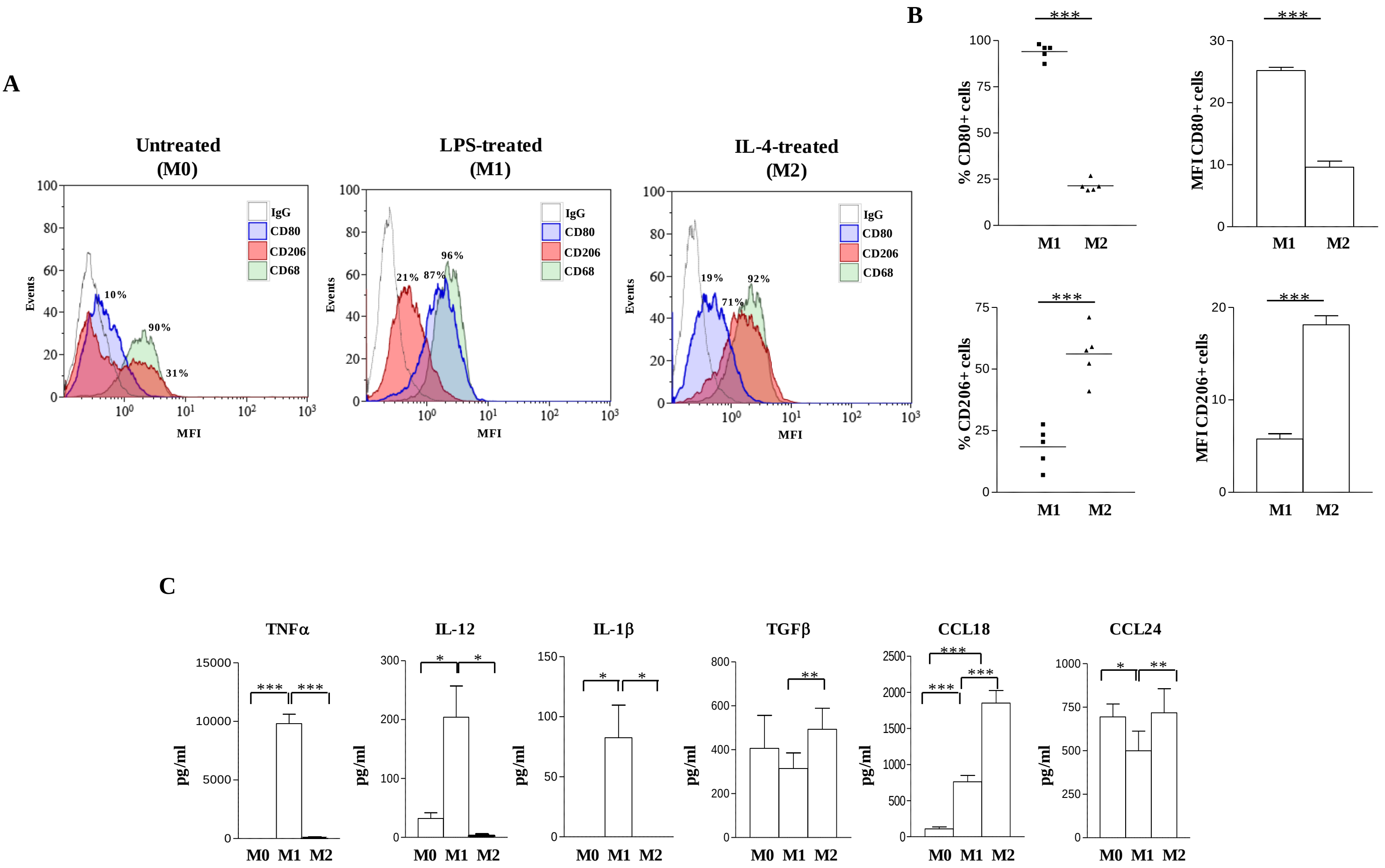

Figure S1. Characterization of phenotypic and secretory features of human M0 unpolarized, M1-, and M2-polarized Mf. Human peripheral blood Mn were differentiated into Mf (M0) by culture for 6 days with M-CSF and then polarized into M1 and M2 subsets by treatment with LPS or IL4, respectively, for additional 24 hr. **(A,B)** Surface marker expression. **(A)** M0, M1-, and M2-polarized Mf were stained with PE-conjugated Abs to CD68, CD80, and CD206 or isotype-matched control Ab and analyzed by flow cytometry. Overlay data are plotted as fluorescence intensity on a log scale versus the number of positive cells. The percentage of positive cells is indicated. Results from a representative donor are shown. **(B)** M1- and M2-polarized Mf were stained with anti-CD80-PE and anti-CD206-PE Abs. *Left panels:* results are presented as a scatter plot and expressed as percentages of positive cells generated from five different donors (dots) corrected for control Ab staining. Horizontal lines represent mean values for each group. *Right panels*: data are shown as a bar graph and expressed as means of the MFI of positive cells ±SEM in five different donors corrected for control Ab staining. *p* values of M1- relative to M2-polarized Mf: ***p<0.001. **(C)** Cytokine secretion**.** Conditioned medium from M0, M1- and M2-polarized Mf was assayed by ELISA to determine indicated cytokine/chemokine content. Results are expressed as pg/8x10^{5} cells/mL and represent the mean ± SEM of three (M0) or five (M1,M2) independent donors. *p* values of M0 relative to M1, M0 relative to M2, and M1 relative to M2; *p<0.05;** p<0.01; ***p<0.001.
