# Supplementary material for: Regulation of Human Macrophage M1–M2 Polarization Balance by Hypoxia and the Triggering Receptor Expressed on Myeloid Cells-1
Source: Front Immunol. 2017 Sep 7;8:1097. doi: 10.3389/fimmu.2017.01097 (PMC5594076; doi:10.3389/fimmu.2017.01097)
Supplement: Supplementary file 2 [file image_2.pdf]

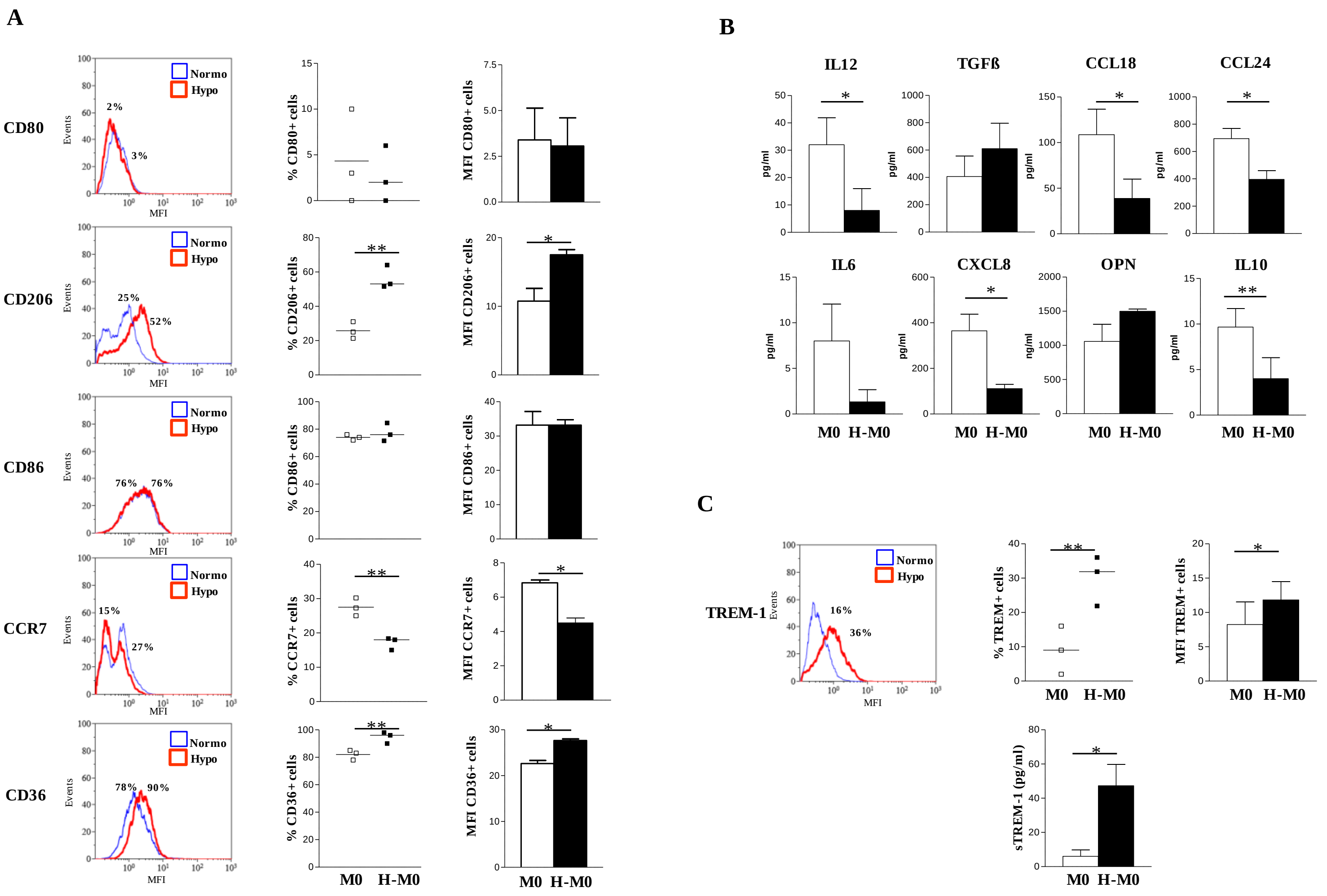


**Figure S2. Characterization of phenotypic and secretory markers of human M0 unpolarized Mf upon differentiation under hypoxia.** Unpolarized Mf were generated as described in the legend of Figure S1 under normoxic or hypoxic condition. **(A)** Surface marker expression. Expression levels of the indicated surface molecules were analyzed by flow cytometry. Histograms depict the results obtained in one representative donor; the blue lines represent the fluorescent profile of normoxic Mf and the red lines represent the fluorescent profile of hypoxic Mf stained with the indicated Abs; the percentage of positive cells is indicated. The scatter plots represent the percentages of positive Mf from three different donors (dots) corrected for control Ab staining. Horizontal lines represent mean values for each group. The bar graphs represent the means of MFI ± SEM of positive cells in three different donors corrected for control Ab staining. *p* values of M0- relative to H-M0-polarized Mf: *p<0.05; **p<0.01. **(B)** Cytokine/chemokine secretion**.** Concentrations of a panel of cytokines/chemokines were assayed by specific ELISA in the supernatant from normoxic (open bars) and hypoxic (full bars) M0 cells analyzed in panel A. Results are expressed as pg/8x10$^{5}$ cells/mL and represent the mean ± SEM of three different experiments. *p* values of M0 relative to H-M0: *p<0.05;** p<0.01. **(C)** TREM-1 surface and secreted levels. *Upper panels:* cells were stained with PE-conjugated anti-TREM-1 Ab, and TREM-1 membrane expression was assessed by flow cytometry. Histograms depict the results obtained from one representative experiments of three performed; positive cell percentages are indicated. The scatter plot represent the percentage of TREM-1-expressing cells from three different donors (dots), with horizontal lines representing mean values for each group. The bar graph represent the MFI ± SEM of TREM-1+ cells in three different donors. p values of M0 relative to H-M0: *p<0.05; **p<0.01. *Lower panel:* sTREM-1 concentrations in cell-free supernatants were measured by ELISA. Results are shown as a bar graph and expressed as pg/8x10$^{5}$ cells/mL. p values of M0 relative to H-M0: * p<0.05.
